# Expression of a heat-stable NADPH-dependent alcohol dehydrogenase in *Caldicellulosiruptor bescii* results in furan aldehyde detoxification

**DOI:** 10.1186/s13068-015-0287-y

**Published:** 2015-07-22

**Authors:** Daehwan Chung, Tobin J Verbeke, Karissa L Cross, Janet Westpheling, James G Elkins

**Affiliations:** BioEnergy Science Center, Oak Ridge National Laboratory, Oak Ridge, TN 37831-6341 USA; Department of Genetics, University of Georgia, Athens, GA 30602-7223 USA; Biosciences Division, Oak Ridge National Laboratory, Oak Ridge, TN 37831-6341 USA

**Keywords:** Thermophile, Pretreatment, Lignocellulose, Biofuel, Butanol dehydrogenase, Furfural, 5-hydroxymethylfurfural, Inhibitor, Genetic engineering, *Caldicellulosiruptor bescii*

## Abstract

**Background:**

Compounds such as furfural and 5-hydroxymethylfurfural (5-HMF) are generated through the dehydration of xylose and glucose, respectively, during dilute-acid pretreatment of lignocellulosic biomass and are also potent microbial growth and fermentation inhibitors. The enzymatic reduction of these furan aldehydes to their corresponding, and less toxic, alcohols is an engineering approach that has been successfully implemented in both *Saccharomyces cerevisiae* and ethanologenic *Escherichia coli*, but has not yet been investigated in thermophiles relevant to biofuel production through consolidated bioprocessing (CBP). Developing CBP-relevant biocatalysts that are either naturally resistant to such inhibitors, or are amenable to engineered resistance, is therefore, an important component in making biofuels production from lignocellulosic biomass feasible.

**Results:**

A butanol dehydrogenase encoding gene from *Thermoanaerobacter pseudethanolicus* 39E (Teth39_1597), previously shown to have furfural and 5-HMF reducing capabilities, was cloned into a suicide plasmid, pDCW171 and transformed into a lactate dehydrogenase mutant of *Caldicellulosiruptor bescii*. Integration of the gene into the *C. bescii* chromosome was verified via PCR amplification and stable expression was observed up to 75°C. Heterologous expression of the NADPH-dependent BdhA enzyme conferred increased resistance of the engineered strain to both furfural and 5-HMF relative to the wild-type and parental strains. Further, when challenged with 15 mM concentrations of either furan aldehyde, the ability to eliminate furfural or 5-HMF from the culture medium was significantly improved in the engineered strain.

**Conclusions:**

A genetically engineered strain of *C. bescii* (JWCB044) has been constructed that shows both an improved tolerance to furan aldehydes and an improved ability to eliminate furfural and 5-HMF from the culture medium. The work presented here represents the first example of engineering furan aldehyde resistance into a CBP-relevant thermophile and further validates *C. bescii* as being a genetically tractable microbe of importance for lignocellulosic biofuel production.

**Electronic supplementary material:**

The online version of this article (doi:10.1186/s13068-015-0287-y) contains supplementary material, which is available to authorized users.

## Background

Several thermophilic, fermentative bacteria that can rapidly solubilize plant-derived polysaccharides are under development as biocatalysts for the low-cost production of biofuels. In contrast to more conventional conversion platforms involving microbes such as *Saccharomyces cerevisiae* or *Zymomonas mobilis*, cellulolytic bacteria including *Clostridium thermocellum* or *Caldicellulosiruptor* spp. already possess the complex molecular machinery necessary to digest lignocellulosic materials through their multifunctional, surface-displayed or free enzymes [1–7]. While the plant deconstruction ability of these organisms is a distinguishing and favorable characteristic, other physiological limitations must be addressed before a thermophilic consolidated bioprocessing (CBP) strategy can be realized. For example, wild-type strains of *Caldicellulosiruptor* spp., the most thermophilic cellulolytic microorganisms so far described (*T*_opt_ ~ 78°C), can simultaneously utilize C5 and C6 sugars and some species have been reported to produce trace amounts of ethanol [8, 9]. Further, advances in genetic and metabolic engineering in *C. bescii* have resulted in improved strains that have fewer fermentation end-products [10] and much higher ethanol yields [11].

Other capacities of *C. bescii* that require further development include improving robustness and tolerance to inhibitory compounds derived from lignocellulosic biomass. Strong inhibitors such as furfural and 5-hydroxymethylfurfural (5-HMF) are generated through the acid-catalyzed dehydration of xylose and glucose, respectively, during the chemical/physical pretreatment of biomass to improve conversion [12]. These aldehydes are known to have a wide range of negative impacts on growth and fermentation for ethanologens including yeast and bacteria [13]. However, tolerance to furfural and 5-HMF in these strains has also been improved through strain evolution and engineering strategies.  For example, in *S. cerevisiae*, expression of ADH6, ADH1, and ARI1 results in the reduction of toxic aldehydes to their less-toxic corresponding alcohols with relatively high activity against furfural and 5-HMF [14–17]. In ethanologenic *Escherichia coli*, furan aldehyde reduction and tolerance have been improved by eliminating an NADPH-dependent alcohol dehydrogenase (*yqhD*) and overexpressing *fucO*, *ucpA* and *pntAB* [18].

*Thermoanaerobacter pseudethanolicus* 39E is an extremely thermophilic, fermentative bacterium that displays tolerance to 20–30 mM concentrations of furan aldehydes and rapidly reduces these compounds in situ during growth [19]. Proteomics analyses have previously shown that a butanol dehydrogenase (BdhA) was upregulated sixfold in response to furfural exposure and suggested the enzyme may be involved in furan aldehyde reduction. Through in vitro studies, it was confirmed that recombinant BdhA could reduce both furfural and 5-HMF using NADPH as the cofactor [19] and the optimal temperature for the enzyme is ~75°C (not shown). In an effort to improve tolerance to furan aldehydes, we attempted to express Teth39_1597 encoding the BdhA enzyme from *T. pseudethanolicus* 39E in an *ldh* mutant (lactate negative) background of *C. bescii*. While multiple gene deletions or additions will likely be necessary for maximum robustness in the presence of furfural and 5-HMF from real-world substrates, this work further demonstrates that *C. bescii* is amenable to strain improvements through rational engineering.

## Results

### Heterologous expression of Teth39_1597 derived from *Thermoanaerobacter pseudethanolicus* 39E in *Caldicellulosiruptor bescii*

**Figure 1:**
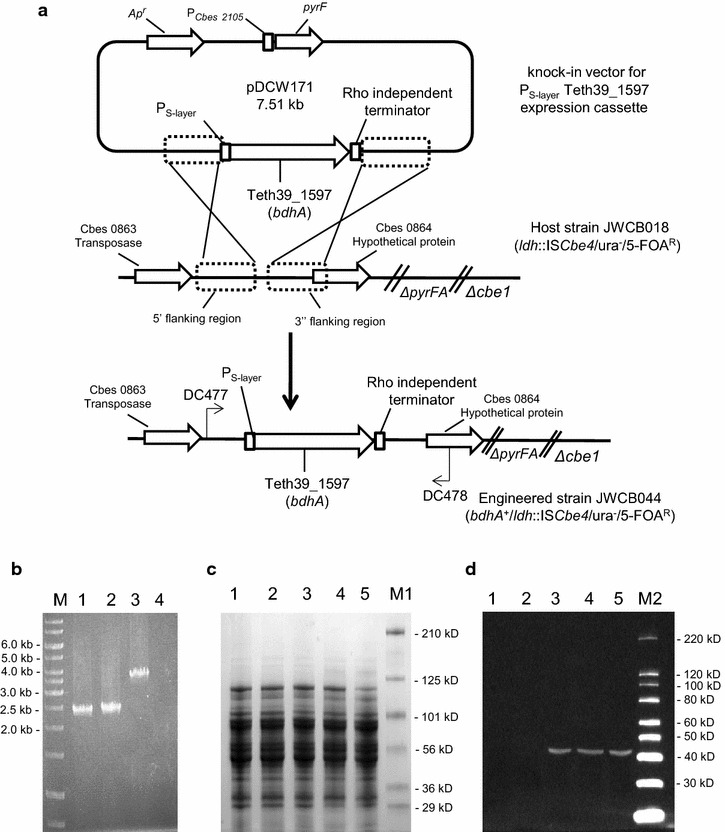
Targeted insertion and expression of the *Thermoanaerobacter pseudethanolicus* 39E *bdhA* gene in *C. bescii*. **a** A diagram of the integration vector pDCW171 (see Additional file 1: Figure S1 for details), which contains the P_S-layer_Teth39_1957 expression cassette and *pyrF* cassette for selection of transformants. Homologous recombination can occur at the upstream or downstream targeted chromosomal regions, integrating the plasmid into the genome and generating a strain that is a uracil prototroph. Counter-selection with 5-FOA selects for loss of the plasmid sequences, but not the Teth39_1957 expression cassette. *Bent arrows* depict primers used for verification of the integrated expression cassette. *Ap* ^*r*^ apramycin resistance gene cassette. **b** Gel depicting PCR products amplified from the targeted chromosome region in JWCB001 (wild-type;* lane 1*), JWCB018 (*ΔpyrFA ldh*::IS*Cbe4 ΔcbeI*;* lane 2*), JWCB044 (*ΔpyrFA ldh*::IS*Cbe4 ΔcbeI* :: *P*_*S*-*layer*_ *Teth39_1597*;* lane 3*), and no DNA (*lane 4*) amplified by primers DC477 and DC478. M: GeneRuler 1 kb DNA ladder (Thermo Scientific). **c**, **d** Total cell protein lysate (80 µg) isolated from mid-log phase cultures were electrophoresed in SDS-PAGE gels either for staining with Coomassie Brilliant Blue (**c**) or for Western blot analysis (**d**) probed with His-tag antibody as described in “Methods”. *Lane 1* JWCB001 grown at 75°C, *lane 2* JWCB018 grown at 75°C, *lane 3* JWCB044 grown at 65°C, *lane 4* JWCB044 grown at 70°C, *lane 5* JWCB044 grown at 75°C, *M1* Pre-stained SDS-PAGE standards, Broad range (Bio-Rad Laboratories) (**c**), *M2* MagicMark™ XP Western Protein Standard (Invitrogen) (**d**).

The BdhA-encoding gene from *T. pseudethanolicus* 39E (Teth39_1597) was cloned and expressed in *C. bescii*. To construct an engineered *C. bescii* strain containing the *T. pseudethanolicus* 39E *bdhA* gene under control of the P_S-layer_ promoter [11], the P_S-layer_Teth39_1597 expression cassette containing a C-terminal 6X His-tag and a Rho-independent transcription terminator was cloned in the suicide vector pDCW171 in *Escherichia coli* (Figure 1a, Additional file 1: Figure S1). This vector also contains a 2.025-kb DNA fragment from the intercistronic region between Cbes0863 and Cbes0864 to allow targeted integration into the *C. bescii* chromosome, and a *pyrF* expression cassette that acts as both a positive and counter-selective marker [11, 20]. The non-replicating vector, pDCW171, was transformed into the uracil auxotroph *C. bescii* lactate dehydrogenase (*ldh*) mutant strain, JWCB018 (*ΔpyrFA ldh*::IS*Cbe4 ΔcbeI*) (Table 1), with selection for uracil prototrophy followed by counter-selection for 5-fluoroorotic acid (5-FOA) resistance, as previously described [21, 22] and depicted in Figure 1a. Initial screening of 20 transformants by PCR revealed merodiploids with a mixture of wild-type and P_S-layer_Teth39_1597 expression cassette insertion genomes. Three of these were further purified on solid medium without 5-FOA and analyzed by PCR amplification using primers DC477 and DC478 to verify a segregated insertion of the expression cassette at the targeted chromosome site. The engineered *C. bescii* strain was designated as JWCB044 (Table 1). As shown in Figure 1b, the parent strain, JWCB018, produced the expected wild-type 2.44 kb band, while amplification from JWCB044 produced a 3.62-kb band indicating a knock-in of the *bdhA* expression cassette within this region. The site of the insertion was confirmed by DNA sequence analysis of the PCR product.

**Table 1:** Strain and plasmids used in this study

Strains	Strain and genotype/phenotype	Source
*Escherichia coli*		
JW334	DH5α containing pDCW171 (Apramycin^R^)	This study
*Caldicellulosiruptor bescii*		
JWCB001	Wild-type/(ura^+^/5-FOA^S^)	DSMZ^a^
JWCB018	*ΔpyrFA ldh*::IS*Cbe4 ΔcbeI*/(ura^−^/5-FOA^R^)	[22, 47]
JWCB044	*ΔpyrFA ldh*::IS*Cbe4 ΔcbeI* :: P_S-layer_Teth39_1597/(ura^−^/5-FOA^R^)	This study
Plasmids		
pET30a-Teth39_1597	Source of Teth39_1597 open reading frame	[11]
pDCW142	Intermediate vector (Apramycin^R^)	[19]
pDCW171	Integration vector containing P_S-layer_Teth39*_*1597 (Apramycin^R^)	This study

^a^Deutsche Sammlung von Mikroorganismen und Zellkulturen.

To investigate the expression and thermo-stability of *T. pseudethanolicus* 39E BdhA in *C. bescii*, JWCB044 was grown in low osmolarity defined (LOD) medium [23] with 5 g/l maltose up to mid-log phase at three temperatures (65, 70 and 75°C). The BdhA protein was difficult to visualize via Coomassie blue staining (Figure 1c), but was clearly visible by Western hybridization analysis using a commercially available His-tag monoclonal antibody (Figure 1d). The 44-kDa size of His-tagged BdhA protein was detected as predicted. The expression levels of the P_S-layer_Teth39_1597 cassettes were similar throughout the tested temperatures (Figure 1d) suggesting that the protein is stable in *C. bescii* up to 75°C.

**Figure 2:**
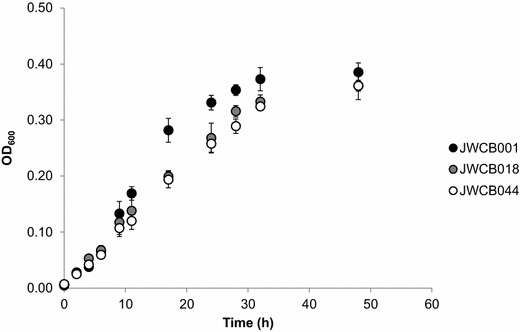
Growth profiles of *C. bescii* wild-type, JWCB018 and JWCB044 in LOD medium at 75°C.

Expression of BdhA had no obvious effect on the growth of JWCB044 relative to the parent strain, JWCB018 (Figure 2). In standard LOD medium in the absence of furan aldehydes, JWCB001, JWCB018 and JWCB044, grew to similar maximal optical densities after 48 h at 75°C. The calculated doubling time of JWCB018 and JWCB044 was ~3.3 h, while that of the wild type was slightly faster at ~3.0 h.

### Strain growth, furan aldehyde resistance and detoxification

**Figure 3:**
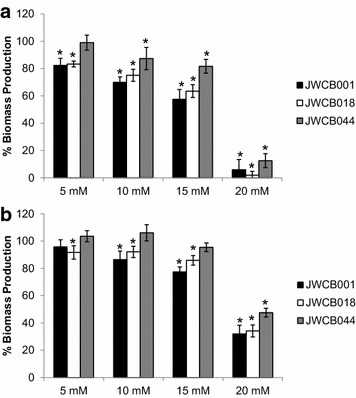
Effect of increasing furfural (**a**) or 5-HMF (**b**) concentrations on biomass production by *C. bescii*. Percent production determined as the average optical density (600 nm) of the experimental condition after 48 h of growth relative to the average optical density of the control condition (0 mM), which represents 100%. The standard error for the control conditions ranged from 4.1 to 5.1%. *Asterisk* designates conditions in which the presence of furfural (**a**) or 5-HMF (**b**) in the culture medium significantly (*p* < 0.05) inhibited growth as determined using a Student’s t test.

To assess if BdhA expression improved strain resistance to furan aldehydes, experiments evaluating the growth of the *C. bescii* strains in the presence of increasing concentrations of furfural or 5-HMF were conducted. Inhibitors were tested at concentrations ranging from 0 to 20 mM, which is consistent with concentrations found in some dilute acid pre-treatment hydrolysates [12, 24, 25] as well as within the inhibitory range determined for the related thermophile, *Caldicellulosiruptor saccharolyticus* [26]. Both the wild-type and JWCB018 strains were significantly (*p* < 0.05) inhibited by furfural at all concentrations tested relative to the 0-mM furfural control condition (Figure 3a, Additional file 2: Figure S2). Similarly, JWCB044 was inhibited at 10, 15 and 20 mM concentrations, but was not significantly inhibited at 5 mM furfural. Despite the observed inhibition, the relative proportion of biomass produced by JWCB044 in the presence of furfural was consistently higher than either the wild-type or the JWCB018 strains.

**Figure 4:**
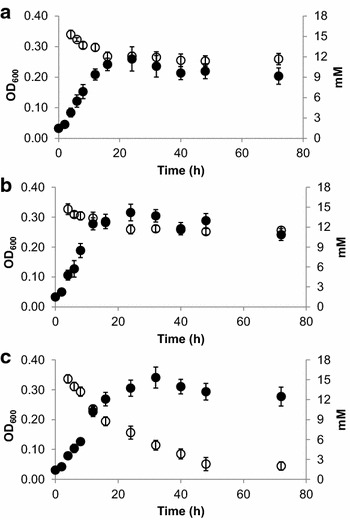
Furfural concentrations relative to growth in *C. bescii* cultures. Furfural (15 mM) was added to LOD medium 4 h after inoculation and residual concentrations were determined via HPLC. **a** Wild-type, **b** JWCB018, **c** JWCB044. *Closed circles* represent the OD_600_, *open circles* represent measured furfural concentrations.

**Figure 5:**
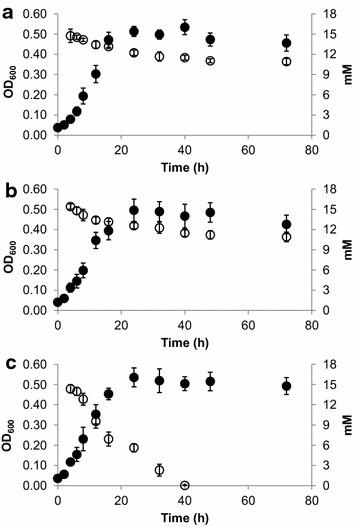
5-Hydroxymethylfurfural (5-HMF) concentrations relative to growth in *C. bescii* cultures. 5-HMF (15 mM) was added to LOD medium 4 h after inoculation and residual concentrations were determined via HPLC. **a** Wild-type, **b** JWCB018, **c** JWCB044. *Closed circles* represent the OD_600_, *open circles* represent measured 5-HMF concentrations. For JWCB044 cultures, the 5-HMF concentrations were below detectable limits at *t* = 48 h and *t* = 72 h.

Increasing concentrations of 5-HMF were similarly found to be inhibitory to all three strains; however, significant inhibition of JWCB044 was only observed at 20 mM 5-HMF (Figure 3b, Additional file 2: Figure S2). All three strains were also generally capable of growth to a greater extent in the presence of 5-HMF relative to growth in the presence of furfural (Figures 4, 5). Presumably, this is due to differences in the toxicity of furfural compared to 5-HMF, with the former exhibiting greater toxic effects as has been reported in other microorganisms [27–30]. The lesser toxicity of 5-HMF relative to furfural is also reflected in the fermentation end-product profiles of the three cultures (Table 2) as concentrations of fermentation end-products were consistently higher for all strains in 5-HMF containing cultures relative to furfural containing cultures.

**Table 2:** Mass balance of 15 mM furfural or 15 mM 5-HMF containing *C. bescii* batch fermentations

	Concentrations (mM)							Carbon Recovery (%)^a^	Concentrations (mM)		O/R^d^
	Cellobiose consumed	Glucose	Acetate	Lactate	CO_2_	Biomass	H_2_		Furfuryl alcohol^b^	5-HMF alcohol^c^	
15 mM Furfural											
JWCB001	6.5 ± 0.6	7.2 ± 0.6	7.3 ± 0.6	3.4 ± 0.3	7.7 ± 0.5	0.4 ± 0.0	12.9 ± 0.9	98.51	3.6 ± 0.8		0.90
JWCB018	5.3 ± 0.3	4.6 ± 0.3	10.5 ± 0.6	ND	10.8 ± 0.8	0.4 ± 0.1	15.5 ± 1.2	95.40	3.2 ± 06		1.11
JWCB044	6.7 ± 0.4	6.0 ± 0.4	14.6 ± 1.0	ND	15.0 ± 0.7	0.5 ± 0.0	18.7 ± 0.7	102.82	13.1 ± 0.6		0.92
15 mM 5-HMF											
JWCB001	10.1 ± 0.5	7.3 ± 0.5	14.4 ± 0.5	9.8 ± 0.9	15.6 ± 0.4	1.2 ± 0.1	24.5 ± 2.5	100.95		3.4 ± 0.4	1.03
JWCB018	9.5 ± 0.3	6.7 ± 0.6	17.8 ± 1.2	ND	18.8 ± 1.5	1.2 ± 0.1	31.9 ± 1.8	87.39		3.9 ± 0.7	1.01
JWCB044	10.1 ± 0.2	7.7 ± 0.7	18.2 ± 1.5	ND	19.4 ± 0.4	1.2 ± 0.2	30.9 ± 1.9	87.57		13.6 ± 0.0	0.84

Metabolite concentrations were determined at the end of 72 h batch fermentations.

*ND* not detected.

^a^Carbon recovery is the ratio of carbon compounds produced divided by the carbon compounds consumed and is expressed as a percentage.

^b^Furfuryl alcohol was not reliably quantifiable via HPLC analyses. Values represent the reduction in furfural from the culture medium and assume 100% reduction to furfuryl alcohol.

^c^Amount of 5-HMF removed from the culture medium was 3.8 ± 0.5 mM (JWCB001), 4.5 ± 0.7 mM (JWCB018) and 14.9 ± 0.0 mM (JWCB044).

^d^Ratio of oxidized end-products to reduced end-products.

As 15 mM furfural was the highest sub-lethal concentration of furfural tested that allowed for growth of all three *C. bescii* cultures, the ability of each strain to eliminate furfural from the growth medium over a 72-h time period was assessed. A decrease of 3.6 ± 0.8 mM furfural was observed in the first 16 h in wild-type cultures (Figure 4a; Table 2), which corresponded to cellular growth phase. After 16 h, significant growth or removal of furfural was not observed. Similar observations were also observed in JWCB018 cultures as both cell growth and furfural removal plateaued after 16 h (Figure 4b). The correlation between growth and inhibitor removal suggests an intrinsic, yet unidentified, ability natively exists in *C. bescii* to reduce furfural and that the decreasing levels observed are not representative of abiotic degradation at elevated temperatures. In JWCB044 cultures, furfural concentrations declined to levels as low as 2.1 ± 0.6 mM after 72 h (Figure 4c). In contrast to the wild-type and JWCB018 cultures, however, levels continued to decline even after cell growth reached maximum levels. The increased removal of furfural from the culture medium by JWCB044 relative to the other strains corresponded to definitive changes in the appearance of the culture medium. After 72 h, JWCB044 cultures had a white, opaque appearance in contrast to the yellow appearance of the wild-type and JWCB018 cultures (data not shown).

Similar observations were also observed when the *C. bescii* cultures were challenged with 15 mM 5-HMF (Figure 5). Significantly more 5-HMF was removed from the culture medium by the JWCB044 cultures than was observed for either the wild-type or JWCB018 strains. After 72 h, 5-HMF levels declined to concentrations of 10.9 ± 0.5 and 10.9 ± 0.7 mM in the wild-type and JWCB018 strains, respectively, whereas the JWCB044 mutant strain removed 5-HMF to below detectable limits.

The addition of furan aldehydes had no obvious effect on carbon flux for any of the three strains tested as mass balance analyses accounted for most of the expected end-products (Table 2). Further, the calculated electron balances suggest that the removal of either furfural or 5-HMF from the culture medium is principally through a reduction reaction where the aldehyde is converted to its corresponding alcohol.

## Discussion

The toxic effects of furan aldehydes formed as a result of dilute acid pretreatment of lignocellulosic biomass represent a major challenge to overcome in developing industry-ready microbes capable of CBP. While the removal of these inhibitory compounds from fermentation broths can be achieved through chemical or physical means [31, 32] these approaches increase process complexity and operating costs. Instead, developing microbes capable of biological inhibitor attenuation, which can occur concurrently with lignocellulose hydrolysis and biofuel production, would be an advantageous approach. As such, the purpose of this study was to engineer this phenotype at, or near the temperature optimum of the CBP-relevant thermophile, *C. bescii* using a furan aldehyde reducing alcohol dehydrogenase.

In the absence of furan aldehydes, both JWCB018 and JWCB044 grew slightly more slowly at 75°C than the wild-type strain, though the growth profiles of the two mutant strains were not significantly different from each other (Figure 2). The slower growth of JWCB018 compared to JWCB001 is consistent with previous reports [11] and exists despite the fact that the *ldh* mutant strain produces more acetate than the wild-type strain, which would lead to an additional ATP per acetate that could be used for biosynthetic processes. Therefore, a possibility exists that this difference could be linked to the removal of a NADH sink in the form of lactate production in JWCB018. Specifically, removal of this electron sink could lead to reduced NAD^+^/NADH co-factor recycling and decrease NAD^+^ levels available for glycolysis, which is considered a key catabolic route in *C. bescii* [10, 33]. In the study by Chung et al. [11], the engineering of an alternative NADH sink in the form of ethanol production restored the growth rate of an *ldh* mutant to the same rate as that of the wild-type strain. Here, the heterologous expression of the *bdhA* gene from *T. pseudethanolicus* 39E did not have the same effect. The fact that growth rate did not increase in the JWCB044 mutant is potentially due to differences in redox balancing. The AdhE from *C. thermocellum* that was previously expressed in *C. bescii* [11] is NADH-linked, while the BdhA-enzyme from *T. pseudethanolicus* 39E is NADPH-dependent [19]. Therefore, instead of restoring growth rate, a potential exists that the combination of engineering approaches employed here could in fact compound possible redox imbalances. Specifically, demands for NADPH could increase through BdhA activity, while NADH could accumulate due to the lack of lactate dehydrogenase activity. However, no enhanced detrimental effect was observed in the JWCB044 strain relative to JWCB018. Further, while both mutant strains grew slightly slower, statistically significant differences in the total biomass produced after 48 h were not observed (Figure 2) suggesting that any potential redox imbalances engineered into the cell have minimal effect on cell viability in the absence of furan aldehydes.

In the presence of furan aldehydes though, the detoxification abilities of JWCB044 led to increased viability and biomass production when cultures were challenged with increasing concentrations of furfural or 5-HMF (Figure 3). At the concentrations tested, 10 mM furfural or 20 mM 5-HMF was needed to significantly (*p* < 0.05) inhibit JWCB044 total biomass production. These concentrations are consistent with what has been reported to be inhibitory in some other fermentative thermophiles including *C. saccharolyticus* [26], *Thermoanaerobacterium thermosaccharolyticum* [34] and *Thermoanaerobacter pentosaceus* [35]. Given that electron disposal does not seem to significantly affect total biomass production in these strains, the improved resistance of JWCB044 is likely due to inhibitor detoxification to the lesser toxic alcohols rather than improved co-factor recycling. This is further supported by the significantly enhanced capabilities of JWCB044 to eliminate furfural or 5-HMF from the culture medium (Figures 4, 5). While previous studies have shown how the addition of external electron acceptors in the form of furan aldehydes can enhance co-factor recycling, and ultimately carbon flux in some strains [36], significant differences in total carbon flux were not observed here (Table 2). Therefore, it is considered likely that the increased resistance of JWCB044 is due to the direct reduction of the furan aldehydes leading to decreased toxicity and not due to resolution of a redox imbalance.

While dilute acid pre-treatment reduces the recalcitrance of lignocellulose, the concomitant formation of inhibitory aldehydes such as furfural and 5-HMF represents a significant obstacle to overcome in making lignocellulosic biofuels through CBP a viable alternative. This makes it necessary to develop inhibitor resistant, industry-ready strains. Additionally, as detoxification typically involves a two-electron reduction of the aldehydes to their corresponding alcohols, furan aldehyde attenuation, therefore, directs electrons away from other reduced, and desirable, products such as ethanol. Rapid elimination of furan aldehydes from culture medium is, therefore, desirable so that reducing power generated through metabolic flux by the microbes can be directly used for biofuel production. In this study, it took *C. bescii* JWCB044 ~ 48 h to completely eliminate 15 mM 5-HMF from the culture medium or for furfural concentrations to reach the lowest concentration observed (Figures 4, 5). In comparison, substantial engineering efforts including deletion of a NADPH-dependent oxidoreductase (*yqh*D) combined with constitutive chromosomal expression of a furfural oxidoreductase-cryptic gene construct (*fucO*-*ucpA*) were required for ethanologenic *E. coli* XW129 to eliminate the same amount of furfural in a 24-h time period [18]. Hence, further engineering efforts may similarly lead to improved rates of furan aldehyde detoxification in *C. bescii*.

A notable advantage of JWCB044 is that when it was challenged with either furfural or 5-HMF, cultures continued to grow and inhibitor removal occurred throughout the growth process. This contrasts with furfural-resistant strains of ethanologenic *E. coli* [18] or *S. cerevisiae* [37], which typically require near-complete removal of the furan aldehyde prior to initiating growth.

## Conclusions

The work presented in this study illustrates successful heterologous expression of BdhA at 75°C in *C. bescii*, which is ~10°C higher than the optimum growth temperature for *T. pseudethanolicus* 39E [38], from where the protein originates, and is significantly higher than previous in vitro studies investigating this enzyme [19]. Its thermostability therefore identifies this protein as having potential utility in other thermophilic hosts being developed for biofuels production from lignocellulosic biomass. Also, this work shows an enhanced capability of engineered *C. bescii* to eliminate furfural or 5-HMF from the culture medium and advances development of this microbe for CBP applications. However, despite this novel phenotype, continued strain development in regard to furan aldehyde detoxification would be beneficial. For example, concentrations in excess of 20 mM furfural or 5-HMF were lethal to JWCB044. These values remain significantly lower than levels tolerated by some engineered yeast strains [39, 40] and are less than the inhibitor concentrations produced under some harsh dilute acid pre-treatment conditions [25, 41]. Achieving tolerance at, or above, currently observed furan aldehyde concentration maxima is desirable. Further, other microbes, such as *T. pseudethanolicus* 39E have a native capacity to reduce comparable levels of furan aldehydes in less than 10 h [19]. Improving cofactor availability, specifically NADPH availability, or finding a means of increasing overall metabolic flux is a possible solution to achieving an increased rate of detoxification. As such, strategies to improve both the rate of reduction and overall tolerance levels will be a matter of ongoing investigation.

## Methods

### Strains, media and culture conditions

*Caldicellulosiruptor bescii* and *E. coli* strains and plasmids used in this study are listed in Table 1. All *C. bescii* strains were grown anaerobically in liquid or on solid surface in LOD medium [23] with maltose or cellobiose (0.5% w/v) as the carbon source, final pH 6.8, supplemented with 40 μM uracil as needed for growth of uracil auxotrophic mutants. Liquid cultures were grown at 75°C in anaerobic culture bottles having undergone iterative degassing: gassing cycles with nitrogen or argon. *E. coli* strain DH5α was used for plasmid DNA constructions and preparations using standard techniques as described [42] and were grown in LB broth supplemented with apramycin (50 μg/mL).

### Vector construction for the knock-in of Teth39_1597 into *C. bescii*

Plasmid DNA from *E. coli* cultures was isolated using a Qiagen Mini-prep Kit (Qiagen, Valencia, CA, USA), while chromosomal DNA from *C. bescii* strains was extracted using the Quick-gDNA™ MiniPrep (Zymo Research, Irvine, CA, USA) or using the DNeasy Blood & Tissue Kit (Qiagen, Valencia, CA, USA) according to the manufacturer’s instructions. A high-fidelity *pfu AD* DNA polymerase (Agilent Technologies, Santa Clara, CA, USA) was used for all PCR reactions, and SphI and PstI restriction enzymes (New England Biolabs, Ipswich, MA, USA), and a Fast-link DNA Ligase kit (Epicentre Biotechnologies, Madison, WI) were used for plasmid construction. Plasmid pDCW171 (Figure 1a, Additional file 1: Figure S1) was constructed by inserting the Teth39_1957 open reading frame into pDCW142 [11], which contains the regulatory region of Cbes2303, a C-terminal 6X Histidine-tag and a Rho-independent transcription terminator. The 6.3-kb DNA fragment was amplified with primers DC466 containing a SphI cut site and DC576 with a PstI cut site using pDCW142 as a template. A 1.2-kb DNA fragment containing the coding sequence of Teth39_1957 was amplified with DC577 containing a PstI site and DC578 containing a SphI site using a previously constructed plasmid (pET30a-Teth39_1597) as template DNA [19]. The two linear DNA fragments were then digested with PstI and SphI and ligated to construct pDCW171 (7.5 kb) (Additional file 1: Figure S1). DNA sequences of the primers used are provided in Additional file 3: Table S1. *E. coli* strain DH5α cells were transformed by electroporation in a 2-mm-gap cuvette at 2.5 V. Apramycin-resistant strains were selected after transformation and the pDCW171 sequences were verified by automatic sequencing (Macrogen USA, Rockville, MD, USA).

### *C. bescii* transformation, screening, purification and sequence verification

To construct strain JWCB044, one microgram of pDCW171 DNA was used to electrotransform JWCB018 (*ΔpyrFA ldh*::IS*Cbe4 ΔcbeI*) as described [21] and cells were plated onto solid LOD medium. Uracil prototrophic transformants were selected and used to inoculate into liquid medium for subsequent genomic DNA extraction and confirmation of the pDCW171 knock-in into the chromosome via PCR screening. Confirmed transformants were inoculated into nonselective liquid defined medium, with 40 μM uracil, and incubated overnight at 75°C to allow loop-out of the plasmid prior to plating onto 5-FOA (8 mM) containing solid medium. After the initial screening, transformants containing the expected knock-in were further purified by one additional passage under selection on solid medium and screened a second time by PCR to check for segregation of the P_S-layer_Teth39_1597 insertion. The insertion of Teth39_1957 at the targeted chromosome region was verified by PCR amplification and sequence analysis using primers DC462 and DC463. A PCR product was generated from genomic DNA using primers DC477 and DC478 targeting outside the homologous regions used to construct the knock-in. All primers and sequences used in this study are listed in Additional file 3: Table S1.

### Preparation of cell lysates and western blotting

Five hundred mL cultures of *C. bescii* were grown to mid-log phase at 65, 70 or 75°C, harvested by centrifugation at 6,000×*g* at 4°C for 15 min and resuspended in Cell-Lytic B cell lysis reagent (Sigma-Aldrich, St. Louis, MO, USA). Cells were lysed by a combination of 4X freeze-thawing and sonication on ice. Protein concentrations were determined using the Bio-Rad protein assay kit (Bio-Rad Laboratories, Hercules, CA, USA) with bovine serum albumin as the standard. Cell free extracts (80 µg) were electrophoresed in 4–15% gradient Mini-Protean TGX gels, which were either stained using Coomassie blue or transferred to PVDF membranes (ImmobilonTM-P; EMD Millipore, Billerica, MA, USA) using a Bio-Rad Mini-Protean 3 electrophoretic apparatus. The membrane was then probed with His-tag (6xHis) monoclonal antibody (1:5,000 dilution; Invitrogen, Grand Island, NY, USA) using the ECL Western Blotting substrate Kit (Thermo Scientific, Waltham, MA, USA) as specified by the manufacturer.

### Physiological characterization and inhibitor detoxification analyses

To determine strain resistance to increasing concentrations of furfural or 5-hydroxymethylfurfural (5-HMF), broth cultures of wild-type *C. bescii*, strain JWCB018 or strain JWCB044 were serially passaged every 24 h in fresh LOD medium containing uracil and 5 g/L cellobiose. After the second transfer, the grown cultures were used to inoculate (5% v/v) Balch tubes containing 9.5 mL of the same medium and supplemented with either furfural or 5-HMF at 0, 5, 10, 15 or 20 concentrations. Growth was monitored by measuring the optical density at 600 nm (OD_600_) routinely for 48 h using a Milton Roy Spectronic 21D spectrophotometer (Milton Roy, Ivyland, PA, USA).

For inhibitor detoxification experiments, batch fermentations were conducted in 125 mL stoppered serum bottles containing 50 mL of medium. The size of the inoculum was varied for each strain such that each culture would have a similar starting OD_600_. For these experiments, the average starting OD_600_ was 0.038 ± 0.008. Post-inoculation, cultures were grown at 75°C for 4 h prior to the addition of either 15 mM furfural or 5-HMF. Throughout the experiment, cell growth was monitored by removing a 1-mL sample from each bottle and measuring the OD_600_ using an Eppendorf BioPhotometer 6131 (Eppendorf, Hamburg, Germany). The 1-mL samples collected were then centrifuged at 14,000×*g* for 5 min and the supernatants analyzed for fermentation end-products, residual furfural and 5-HMF levels and furan alcohol formation. Soluble analytes were measured via high-performance liquid chromatography (HPLC) using a Waters Breeze 2 system (Waters Corp., Milford, MA, USA) equipped with a refractive index detector (model 2414) and an Aminex HPX-87H column (Bio-Rad Laboratories). Sulfuric acid (5 mM) was used as the mobile phase at a flow rate of 0.6 ml/min. Peak areas and retention times were compared against known standards of the same analyte.

Hydrogen in the headspace was measured using an Agilent Technologies 6850 Series II gas chromatograph with a Carboxen 1010 plot column (30 m × 0.53 mm) and a thermal conductivity detector set at 190°C. Hydrogen in the liquid fraction was determined as previously described [43] accounting for the gas solubility constant [44]. Carbon dioxide measurements were estimated and represent the sum of the determined values for acetate and biomass production. Protein/biomass measurements were also conducted as previously described [43], whereby total cellular protein was determined via a standard Bradford assay and cellular protein in *C. bescii* is assumed to be 50% of the cell dry weight [45]. An elemental composition of C_4_H_7_O_2_N was used for biomass determinations in moles of carbon [46]. Reported values for all physiological characterization experiments are averages calculated from two independent experiments with each experiment including three biological replicates.

## Additional file

Additional file 1:
**Figure S1**. Diagram of the Teth39_1597 expression cassette integration vector. Apr, apramycin resistant gene cassette; pSC101, low copy replication origin in *E. coli*; repA, a plasmid-encoded gene required for pSC101 replication; *par*, partition locus. Two kb flanking regions used for homologous recombination and a *pyrF* cassette for selection of uracil prototrophy are indicated. The *bdhA* (Teth39_1597) expression cassette is indicated. Additional file 2:
**Figure S2**. Growth profiles in the presence of increasing furan aldehyde concentrations. Strain JWCB001 (A, B), JWCB018 (C,D), and JWCB044 (E,F). Additional file 3:
**Table S1**. Primers used in this study.

## Abbreviations

5-FOA: 5-fluoroorotic
5-HMF: 5-hydroxymethylfurfural
BDH: butanol dehydrogenase
CBP: consolidated bioprocessing
HPLC: high-performance liquid chromatography
LDH: lactate dehydrogenase
LOD: low osmolarity defined
OD: optical density

## References

[1] Bayer EA, Morag E, Lamed R (1994). The cellulosome—a treasure-trove for biotechnology. Trends Biotechnol.

[2] Bayer EA, Belaich JP, Shoham Y, Lamed R (2004). The cellulosomes: multienzyme machines for degradation of plant cell wall polysaccharides. Annu Rev Microbiol.

[3] Dworkin M, Falkow S, Rosenberg E, Schleifer K-H, Stackebrandt E, Bayer E (2006). Cellulose-decomposing bacteria and their enzyme systems. The Prokaryotes.

[4] Shoham Y, Lamed R, Bayer EA (1999). The cellulosome concept as an efficient microbial strategy for the degradation of insoluble polysaccharides. Trends Microbiol.

[5] Himmel ME, Xu Q, Luo Y, Ding S-Y, Lamed R, Bayer EA (2010). Microbial enzyme systems for biomass conversion: emerging paradigms. Biofuels.

[6] Brunecky R, Alahuhta M, Xu Q, Donohoe BS, Crowley MF, Kataeva IA (2013). Revealing nature’s cellulase diversity: the digestion mechanism of *Caldicellulosiruptor bescii* CelA. Science.

[7] Blumer-Schuette SE, Giannone RJ, Zurawski JV, Ozdemir I, Ma Q, Yin YB (2012). *Caldicellulosiruptor* core and pangenomes reveal determinants for noncellulosomal thermophilic deconstruction of plant biomass. J Bacteriol.

[8] Rainey FA, Donnison AM, Janssen PH, Saul D, Rodrigo A, Bergquist PL (1994). Description of *Caldicellulosiruptor saccharolyticus* gen. nov., sp. nov: an obligately anaerobic, extremely thermophilic, cellulolytic bacterium. FEMS Microbiol Lett.

[9] Hamilton-Brehm SD, Mosher JJ, Vishnivetskaya T, Podar M, Carroll S, Allman S (2010). *Caldicellulosiruptor obsidiansis* sp nov., an anaerobic, extremely thermophilic, cellulolytic bacterium isolated from Obsidian Pool, Yellowstone National Park. Appl Environ Microbiol.

[10] Cha M, Chung DW, Elkins JG, Guss AM, Westpheling J (2013). Metabolic engineering of *Caldicellulosiruptor bescii* yields increased hydrogen production from lignocellulosic biomass. Biotechnol Biofuels.

[11] Chung D, Cha M, Guss AM, Westpheling J (2014). Direct conversion of plant biomass to ethanol by engineered *Caldicellulosiruptor bescii*. Proc Natl Acad Sci USA.

[12] Guo G-L, Chen W-H, Chen W-H, Men L-C, Hwang W-S (2008). Characterization of dilute acid pretreatment of silvergrass for ethanol production. Bioresour Technol.

[13] Jonsson LJ, Alriksson B, Nilvebrant N-O (2013). Bioconversion of lignocellulose: inhibitors and detoxification. Biotechnol Biofuels.

[14] Liu ZL (2011). Molecular mechanisms of yeast tolerance and in situ detoxification of lignocellulose hydrolysates. Appl Microbiol Biotechnol.

[15] Almeida JRM, Modig T, Petersson A, Hahn-Hagerdal B, Liden G, Gorwa-Grauslund MF (2007). Increased tolerance and conversion of inhibitors in lignocellulosic hydrolysates by *Saccharomyces cerevisiae*. J Chem Technol Biotechnol.

[16] Petersson A, Almeida JRM, Modig T, Karhumaa K, Hahn-Hagerdal B, Gorwa-Grauslund MF (2006). A 5-hydroxymethyl furfural reducing enzyme encoded by the *Saccharomyces cerevisiae* ADH6 gene conveys HMF tolerance. Yeast.

[17] Liu ZL, Moon J (2009). A novel NADPH-dependent aldehyde reductase gene from *Saccharomyces cerevisiae* NRRL Y-12632 involved in the detoxification of aldehyde inhibitors derived from lignocellulosic biomass conversion. Gene.

[18] Wang X, Yomano LP, Lee JY, York SW, Zheng H, Mullinnix MT (2013). Engineering furfural tolerance in *Escherichia coli* improves the fermentation of lignocellulosic sugars into renewable chemicals. Proc Natl Acad Sci USA.

[19] Clarkson SM, Hamilton-Brehm SD, Giannone RJ, Engle NL, Tschaplinski TJ, Hettich RL (2014). A comparative multidimensional LC-MS proteomic analysis reveals mechanisms for furan aldehyde detoxification in *Thermoanaerobacter pseudethanolicus* 39E. Biotechnol Biofuels.

[20] Chung D, Cha M, Farkas J, Westpheling J (2013). Construction of a stable replicating shuttle vector for *Caldicellulosiruptor* species: use for extending genetic methodologies to other members of this genus. Plos One.

[21] Chung D, Farkas J, Huddleston JR, Olivar E, Westpheling J (2012). Methylation by a unique α-class N_4_-cytosine methyltransferase is required for dna transformation of *Caldicellulosiruptor bescii* DSM6725. PLoS One.

[22] Chung DW, Farkas J, Westpheling J (2013). Overcoming restriction as a barrier to DNA transformation in *Caldicellulosiruptor* species results in efficient marker replacement. Biotechnol Biofuels.

[23] Farkas J, Chung D, Cha M, Copeland J, Grayeski P, Westpheling J (2013). Improved growth media and culture techniques for genetic analysis and assessment of biomass utilization by *Caldicellulosiruptor bescii*. J Ind Microbiol Biotechnol.

[24] Hsu T-C, Guo G-L, Chen W-H, Hwang W-S (2010). Effect of dilute acid pretreatment of rice straw on structural properties and enzymatic hydrolysis. Bioresour Technol.

[25] Redding AP, Wang Z, Keshwani DR, Cheng JJ (2011). High temperature dilute acid pretreatment of coastal Bermuda grass for enzymatic hydrolysis. Bioresour Technol.

[26] de Vrije T, Bakker RR, Budde MAW, Lai MH, Mars AE, Claassen PAM (2009). Efficient hydrogen production from the lignocellulosic energy crop *Miscanthus* by the extreme thermophilic bacteria *Caldicellulosiruptor saccharolyticus* and *Thermotoga neapolitana*. Biotechnol Biofuels.

[27] Zaldivar J, Martinez A, Ingram LO (1999). Effect of selected aldehydes on the growth and fermentation of ethanologenic *Escherichia coli*. Biotechnol Bioeng.

[28] Taherzadeh MJ, Gustafsson L, Niklasson C, Liden G (2000). Physiological effects of 5-hydroxymethylfurfural on *Saccharomyces cerevisiae*. Appl Microbiol Biotechnol.

[29] Franden MA, Pienkos PT, Zhang M (2009). Development of a high-throughput method to evaluate the impact of inhibitory compounds from lignocellulosic hydrolysates on the growth of *Zymomonas mobilis*. J Biotechnol.

[30] Quemeneur M, Hamelin J, Barakat A, Steyer J-P, Carrere H, Trably E (2012). Inhibition of fermentative hydrogen production by lignocellulose-derived compounds in mixed cultures. Int J Hydrogen Energy.

[31] Millati R, Niklasson C, Taherzadeh MJ (2002). Effect of pH, time and temperature of overliming on detoxification of dilute-acid hydrolyzates for fermentation by *Saccharomyces cerevisiae*. Process Biochem.

[32] Gurram RN, Datta S, Lin YJ, Snyder SW, Menkhaus TJ (2011). Removal of enzymatic and fermentation inhibitory compounds from biomass slurries for enhanced biorefinery process efficiencies. Bioresour Technol.

[33] Carere CR, Rydzak T, Verbeke TJ, Cicek N, Levin DB, Sparling R (2012). Linking genome content to biofuel production yields: a meta-analysis of major catabolic pathways among select H_2_ and ethanol-producing bacteria. BMC Microbiol.

[34] Cao GL, Ren NQ, Wang AJ, Guo WQ, Xu JF, Liu BF (2010). Effect of lignocellulose-derived inhibitors on growth and hydrogen production by *Thermoanaerobacterium thermosaccharolyticum* W16. Int J Hydrogen Energy.

[35] Tomas AF, Karagoz P, Karakashev D, Angelidaki I (2013). Extreme thermophilic ethanol production from rapeseed straw: using the newly isolated *Thermoanaerobacter pentosaceus* and combining it with *Saccharomyces cerevisiae* in a two-step process. Biotechnol Bioeng.

[36] Zhang Y, Han B, Ezeji TC (2012). Biotransformation of furfural and 5-hydroxymethyl furfural (HMF) by *Clostridium acetobutylicum* ATCC 824 during butanol fermentation. New Biotechnol.

[37] Liu ZL, Ma MG, Song MZ (2009). Evolutionarily engineered ethanologenic yeast detoxifies lignocellulosic biomass conversion inhibitors by reprogrammed pathways. Mol Genet Genomics.

[38] Onyenwoke RU, Kevbrin VV, Lysenko AM, Wiegel J (2007). *Thermoanaerobacter pseudethanolicus* sp. nov., a thermophilic heterotrophic anaerobe from Yellowstone National Park. Int J Syst Evol Microbiol.

[39] Gorsich SW, Dien BS, Nichols NN, Slininger PJ, Liu ZL, Skory CD (2006). Tolerance to furfural-induced stress is associated with pentose phosphate pathway genes ZWF1, GND1, RPE1, and TKL1 in *Saccharomyces cerevisiae*. Appl Microbiol Biotechnol.

[40] Heer D, Heine D, Sauer U (2009). Resistance of *Saccharomyces cerevisiae* to high concentrations of furfural is based on NADPH-dependent reduction by at least two oxireductases. Appl Environ Microbiol.

[41] Taherzadeh MJ, Niklasson C, Liden G (1999). Conversion of dilute-acid hydrolyzates of spruce and birch to ethanol by fed-batch fermentation. Bioresour Technol.

[42] Sambrook J, Russell DW (2001). Molecular cloning. A laboratory manual.

[43] Verbeke TJ, Spicer V, Krokhin OV, Zhang X, Schellenberg JJ, Fristensky B (2014). *Thermoanaerobacter thermohydrosulfuricus* WC1 shows protein complement stability during fermentation of key lignocellulose-derived substrates. Appl Environ Microbiol.

[44] Sander R (1999) Compilation of Henry’s law constants for inorganic and organic species of potential importance in environmental chemistry. Max-Planck Institute of Chemistry, Air Chemistry Department Mainz, Germany

[45] Basen M, Rhaesa AM, Kataeva I, Prybol CJ, Scott IM, Poole FL (2014). Degradation of high loads of crystalline cellulose and of unpretreated plant biomass by the thermophilic bacterium *Caldicellulosiruptor bescii*. Bioresour Technol.

[46] Guedon E, Payot S, Desvaux M, Petitdemange H (1999). Carbon and electron flow in *Clostridium cellulolyticum* grown in chemostat culture on synthetic medium. J Bacteriol.

[47] Cha M, Wang H, Chung D, Bennetzen JL, Westpheling J (2013). Isolation and bioinformatic analysis of a novel transposable element, IS*Cbe4*, from the hyperthermophilic bacterium, *Caldicellulosiruptor bescii*. J Ind Microbiol Biotechnol.

