# Supplementary material for: Expression of a heat-stable NADPH-dependent alcohol dehydrogenase in Caldicellulosiruptor bescii results in furan aldehyde detoxification
Source: Biotechnol Biofuels. 2015 Jul 22;8:102. doi: 10.1186/s13068-015-0287-y (PMC4511240; doi:10.1186/s13068-015-0287-y)
Supplement: Additional file 1: — Figure S1. Diagram of the Teth39_1597 expression cassette integration vector. Apr, apramycin resistant gene cassette; pSC101, low copy replication origin in E. coli; repA, a plasmid-encoded gene required for pSC101 replication; par, partition locus. Two kb flanking regions used for homologous recombination and a pyrF cassette for selection of uracil prototrophy are indicated. The bdhA (Teth39_1597) expression cassette is indicated. [file 13068_2015_287_MOESM1_ESM.pdf]

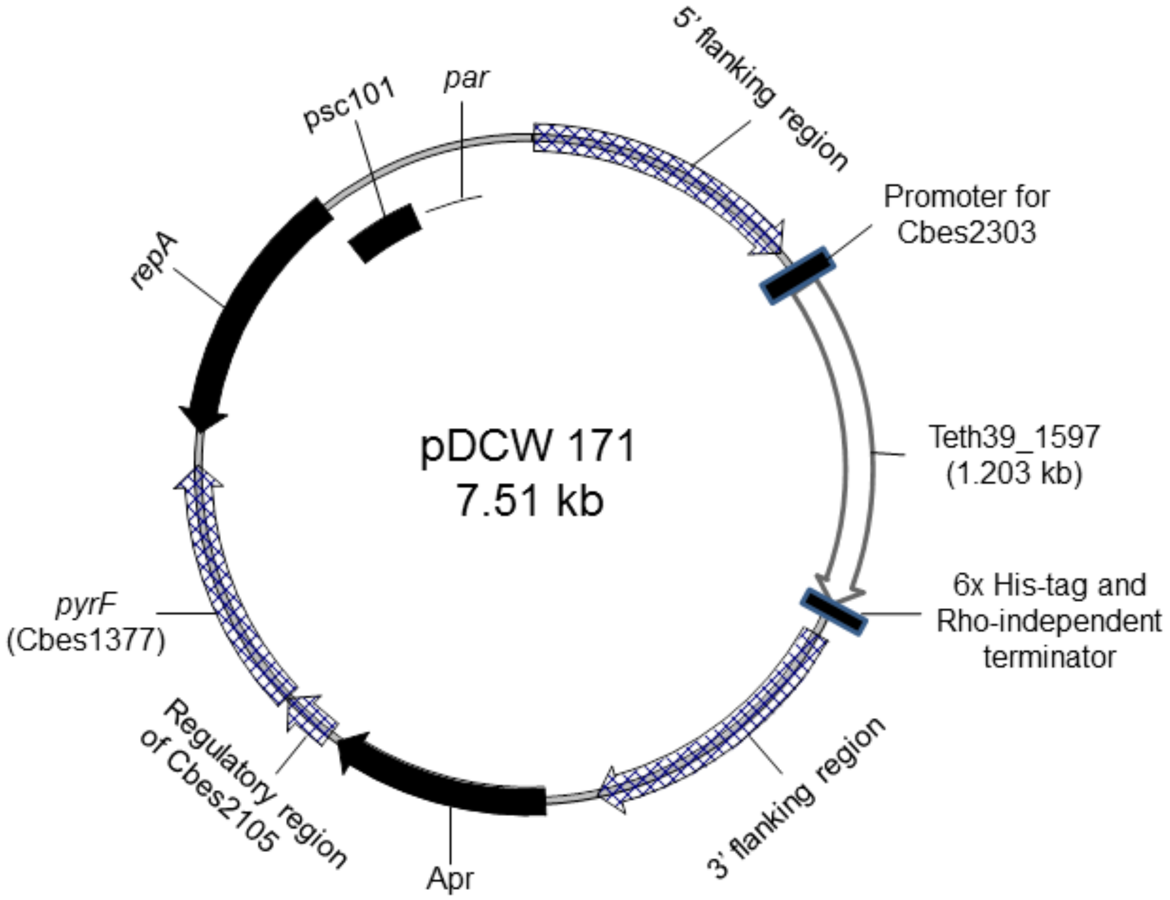


**Figure S1. Diagram of the Teth39_1597 expression cassette integration vector.** Apr, apramycin resistant gene cassette; pSC101, low copy replication origin in *E. coli*; *repA*, a plasmid-encoded gene required for pSC101 replication; *par*, partition locus. Two kb flanking regions used for homologous recombination and a *pyrF* cassette for selection of uracil prototrophy are indicated. The *bdhA* (Teth39_1597) expression cassette is indicated.
