# Supplementary material for: Expression of a heat-stable NADPH-dependent alcohol dehydrogenase in Caldicellulosiruptor bescii results in furan aldehyde detoxification
Source: Biotechnol Biofuels. 2015 Jul 22;8:102. doi: 10.1186/s13068-015-0287-y (PMC4511240; doi:10.1186/s13068-015-0287-y)
Supplement: Additional file 2: — Figure S2. Growth profiles in the presence of increasing furan aldehyde concentrations. Strain JWCB001 (A, B), JWCB018 (C,D), and JWCB044 (E,F). [file 13068_2015_287_MOESM2_ESM.pdf]

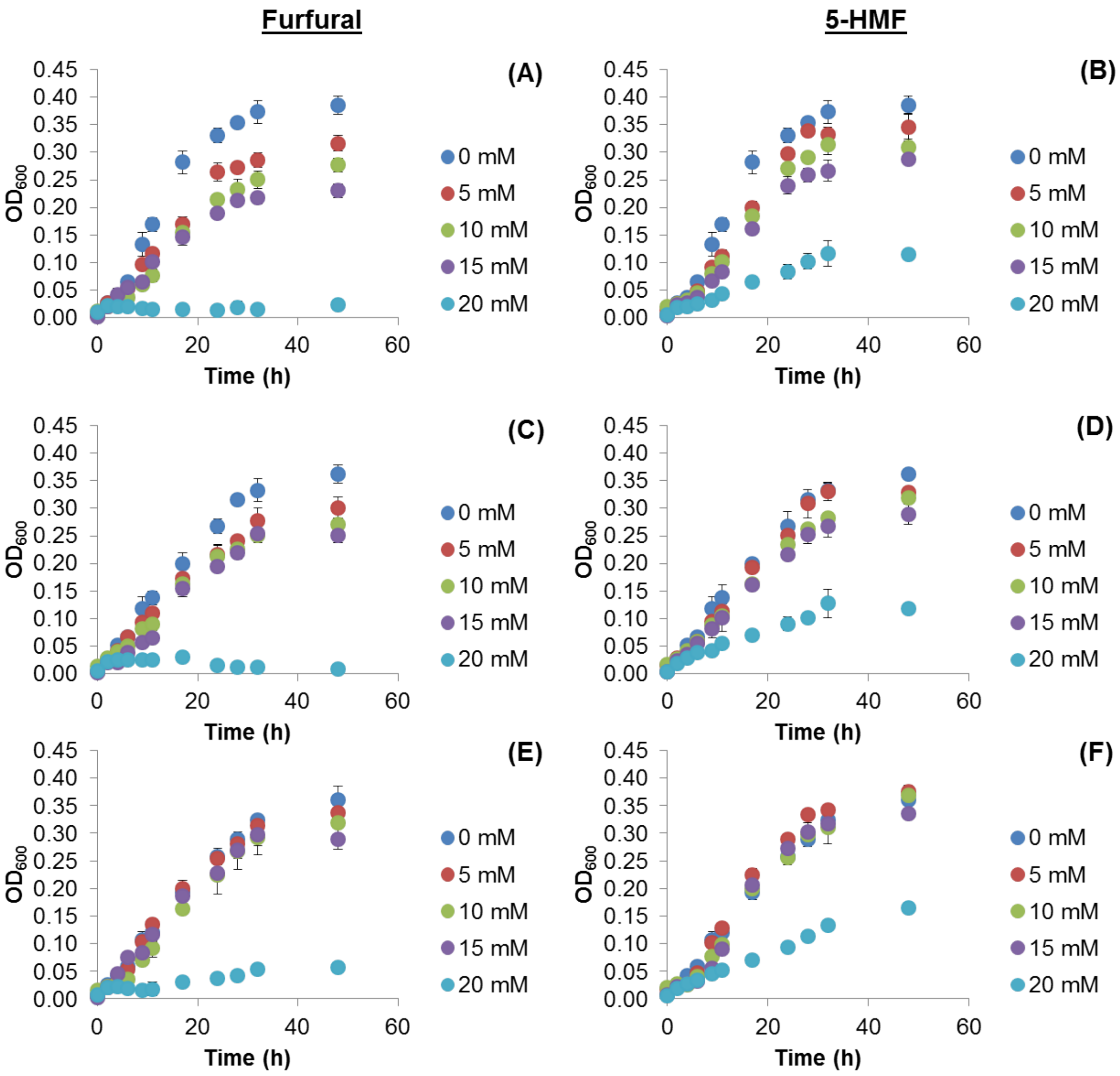

Figure S2. Growth profiles in the presence of increasing furan aldehyde concentrations. Strain JWCB001 (A, B), JWCB018 (C,D), and JWCB044 (E,F).
