# Supplementary material for: Expression of a heat-stable NADPH-dependent alcohol dehydrogenase in Caldicellulosiruptor bescii results in furan aldehyde detoxification
Source: Biotechnol Biofuels. 2015 Jul 22;8:102. doi: 10.1186/s13068-015-0287-y (PMC4511240; doi:10.1186/s13068-015-0287-y)
Supplement: Additional file 3: — Table S1. Primers used in this study. [file 13068_2015_287_MOESM3_ESM.pdf]

Table S1 Primers used in this study.

Primers	Sequences (5’ to 3’)	Description
DC462	TGCTGGCAGAGAAGAGCGAAA	To verify the targeted insertion of $P_{S\text{-}layer}$Teth39_1597
DC463	TCTTCATCCCAATCTTCAACTTC	To verify the targeted insertion of $P_{S\text{-}layer}$Teth39_1597
DC466	AGAGCATGCCATCACCATCACCATCACTAATAATAAAGCTGAAATAAAAGAGGGTGAGA	To construct pDCW171
DC477	TGGTTGACCAGGAGAATTTTACACA	To verify the targeted insertion of $P_{S\text{-}layer}$Teth39_1597
DC478	AGCAACAATCCTGCATTTGTAAG	To verify the targeted insertion of $P_{S\text{-}layer}$Teth39_1597
DC576	ACTACTCTGCAGCTCACCAAACCTCCTTGTATGAT	To construct pDCW171
DC577	ACTCTGCAGATGATGAAATTTGAATTTTATAACCCGACC	To construct pDCW171
DC578	AGAGCATGCCATTGCCATGCGTAGTATTTCTA	To construct pDCW171
